# Effects of indoor residual spraying and outdoor larval control on *Anopheles coluzzii* from São Tomé and Príncipe, two islands with pre-eliminated malaria

**DOI:** 10.1186/s12936-019-3037-y

**Published:** 2019-12-05

**Authors:** Ying-An Chen, Jih-Ching Lien, Lien-Fen Tseng, Chien-Fu Cheng, Wan-Yu Lin, Hurng-Yi Wang, Kun-Hsien Tsai

**Affiliations:** 1 Institute of Environmental and Occupational Health Sciences, College of Public Health, National Taiwan University, Taipei, Taiwan; 2 Taiwan Anti-Malaria Advisory Mission, São Tomé, São Tomé and Príncipe; 3 Institute of Epidemiology and Preventive Medicine, College of Public Health, National Taiwan University, Taipei, Taiwan; 4 Department of Public Health, College of Public Health, National Taiwan University, Taipei, Taiwan; 5 Graduate Institute of Clinical Medicine, College of Medicine, National Taiwan University, Taipei, Taiwan

**Keywords:** São Tomé and Príncipe, *Anopheles coluzzii*, Indoor residual spraying, *Bacillus thuringiensis israelensis*, Vector density, Cytochrome *c* oxidase subunit I, Knockdown resistance mutation

## Abstract

**Background:**

Vector control is a key component of malaria prevention. Two major vector control strategies have been implemented in São Tomé and Príncipe (STP), indoor residual spraying (IRS) and outdoor larval control using *Bacillus thuringiensis israelensis* (*Bti*). This study evaluated post-intervention effects of control strategies on vector population density, composition, and knockdown resistance mutation, and their implications for malaria epidemiology in STP.

**Methods:**

Mosquitoes were collected by indoor and outdoor human landing catches and mosquito light traps in seven districts. Mosquito density was calculated by numbers of captured adult mosquitoes/house/working hour. Mitochondrial cytochrome *c* oxidase subunit I (*COI*) was PCR amplified and sequenced to understand the spatial–temporal population composition of malaria vector in STP. Knockdown resistance L1014F mutation was detected using allele-specific PCR. To estimate the malaria transmission risks in STP, a negative binomial regression model was constructed. The response variable was monthly incidence, and the explanatory variables were area, rainfall, entomological inoculation rate (EIR), and *kdr* mutation frequency.

**Results:**

Malaria vector in STP is exophilic *Anopheles coluzzii* with significant population differentiation between Príncipe and São Tomé (mean F_ST_ = 0.16, p < 0.001). Both vector genetic diversity and knockdown resistance mutation were relatively low in Príncipe (mean of *kdr* frequency = 15.82%) compared to São Tomé (mean of *kdr* frequency = 44.77%). Annual malaria incidence rate in STP had been rapidly controlled from 37 to 2.1% by three rounds of country-wide IRS from 2004 to 2007. Long-term application of *Bti* since 2007 kept the mosquito density under 10 mosquitoes/house/hr/month, and malaria incidence rate under 5% after 2008, except for a rising that occurred in 2012 (incidence rate = 6.9%). Risk factors of area (São Tomé compared to Príncipe), rainfall, outdoor EIR, and *kdr* mutation frequency could significantly increase malaria incidence by 9.33–11.50, 1.25, 1.07, and 1.06 fold, respectively.

**Conclusions:**

Indoor residual spraying could rapidly decrease *Anopheles* density and malaria incidence in STP. Outdoor larval control using *Bti* is a sustainable approach for controlling local vector with exophilic feature and insecticide resistance problem. Vector control interventions should be intensified especially at the north-eastern part of São Tomé to minimize impacts of outbreaks.

## Background

Malaria, a disease caused by *Plasmodium* parasites transmitted by *Anopheles* species, was responsible for 219 million malaria cases and 435 thousand deaths globally in 2017 [1]; about 90% of malaria cases and deaths occurred in Africa. The dominant malaria vectors in Africa belong to the *Anopheles gambiae* complex or the *Anopheles funestus* complex, which have long lifespans and strong human-biting habits [2].

São Tomé and Príncipe (STP) is an island nation located in the Gulf of Guinea, Central West Africa. It mainly consists of two islands, São Tomé main island and Príncipe offshore island. The reported malaria vector in STP is *Anopheles gambiae* M form [3–5], also known as *Anopheles coluzzii* [6], a cryptic species mainly found in West Africa belonging to the *An. gambiae* complex [7]. Another *Anopheles* species that can be found in STP is *Anopheles coustani* [3], but it is not considered responsible for the transmission of local malaria due to its small population. Unlike the African continent which is inhabited by various vector species [2, 8], malaria vector species in STP is less diverse and more unique due to its geographic isolation. Previous studies used genetic markers such as mitochondrial NADH dehydrogenase subunit 5 (*ND5*), rDNA intergenic spacer (*IGS*) and internal transcribed spacer, microsatellite DNA, and transposable elements for understanding the origins and structures of *An. coluzzii*, and the possibilities of implementing transgenic technologies in STP [4, 5, 9, 10]. Recently, DNA barcoding by cytochrome *c* oxidase subunit I (*COI*) has gained increasing popularity due to its ease of amplification, high copy number, lack of recombination, and constant evolutionary rate [11, 12]. It can be used to identify species, estimate phylogenies among closely related taxa, and trace evolutionary history [13]. It is especially beneficial for identifying cryptic species that are difficult to distinguish by morphology. For example, *COI* was used to reconstruct the molecular phylogeny of cryptic species members in *Anopheles hyrcanus* group in Asia [14], and *Anopheles albitarsis* complex in South America [15]. Thus, *COI* has become a popular and basic marker for understanding the species composition in vector mosquitoes. This study aims to explore the vector population composition by comparing *COI* sequences in *Anopheles* mosquitoes within STP, and with those from other African continental countries.

In the early 1980s, a national control programme spraying DDT in STP failed to reduce malaria incidence and effective population size of vector [16, 17]. In response to the United Nations Millennium Development Goal 6 during 2000 to 2015, Taiwan Anti-Malaria Advisory Mission collaborated with Centro National de Endemias of STP to scale up the national malaria prevention programme since 2004 [18]. During 2004 to 2006, implementation of IRS by alpha-cypermethrin successfully decreased the prevalence of malaria parasitaemia in children under 9-years-old from 20.1 to 0.6% [19]. In 2007, integrated vector management (IVM) was also implemented. IVM takes ecological and epidemiological characteristics of vectors into consideration [20], and the programme did so by applying an alternative vector control strategy using the microorganism *Bacillus thuringiensis israelensis* (*Bti*) to kill mosquito larvae. Both early and late instars of *Anophele*s larvae immediately responded to *Bti* application and reached 100% mortality in the laboratory and field [21, 22]. Other control strategies implemented in STP are intermittent preventive treatment in pregnancy since 2004, distribution of long-lasting insecticidal nets (LLINs) and the use of artemisinin-based combination therapy (ACT) since 2005, and mass-screening by rapid diagnostic test since 2008 [18, 23]. Due to the success of these control strategies, STP is on track for a 20–40% reduction in incidence by 2020, which is one of the targets in World Health Organization (WHO) Global Technical Strategy for Malaria 2016–2030 [24]. Furthermore, Príncipe island has reached the criterion of malaria pre-elimination [25]. The path to eliminating malaria should be promising under the premise of continued long-term follow-ups. Longitudinal monitoring of vector dynamic changes post-interventions and challenges such as potential insecticide resistance would be addressed in this study.

Severe chemical insecticide resistance is a major public health issue in many countries. According to WHO’s Global Report on Insecticide Resistance in Malaria Vectors: 2010–2016 [26], 68 countries have reported resistance to at least one class of insecticide, 57 of which reported resistance to two or more. Pyrethroid is the most commonly used insecticide class in IRS and insecticide-treated nets (ITNs). The L1014F and L1014S replacements are the major knockdown resistance mutations in *An. gambiae* sensu lato (*s.l*.) [27]. *Anopheles coluzzii* is a species that originally carried low *kdr* L1014F mutation (6.3–40%) before 2006 in West Africa [28]. However, after the year of 2010, there have been several reports on the development of high frequency of the *kdr* L1014F mutation (over 80–90%) showing up in *An. coluzzii* from Central and West Africa [29–33]. Although alpha-cypermethrin has shown a significant prevention effect against malaria transmission in STP, excessive use of insecticide can result in serious insecticide resistance problem. Since the insecticide resistance data has not been systematically surveyed in STP, this study aimed to conduct a longitudinal and country-wide survey on *kdr* in the local malaria vector collected from 2010 to 2016 to clarify the pyrethroid resistance status and its impact on malaria transmission in STP.

Malaria control and elimination in geographically isolated islands are less complicated compared to continental areas [34]. It is important to understand the transmission patterns on this isolated island nation for eliminating malaria. This study focused on investigating the characteristics of malaria vector and its implications for malaria epidemiology in STP. Spatial and temporal variations of vector density, population genetics, knockdown resistance mutation, and their potential risks to malaria epidemics form the core of this study.

## Methods

### Ethics statement

The content and methods used in this study have been reviewed and approved by the Centro Nacional de Endemias of the Ministry of Health in STP (official No. OF^0^N^0^19/P^0^CNE/2016) and the Research Ethics Committee of National Taiwan University Hospital (NTUH-REC No.: 201110023RD).

### Study site

São Tomé and Príncipe islands are both located on the Cameroon Volcanic Line separated by 150 km of ocean. The total area is approximately 1001 km^2^ inhabited by nearly 200 thousand residents (source: Wikipedia- https://en.wikipedia.org/wiki/S%C3%A3o_Tom%C3%A9_and_Pr%C3%ADncipe). Príncipe is an autonomous region where only 5% of the total population resides. There are six administrative districts in São Tomé, Água Grande (AG), Mé-Zóchi (MZ), Lobata (LO), Cantagalo (CT), Lembá (LE), and Caué (CU). With volcanic mountains distributed in southwest of São Tomé from Lembá to Caué, population are concentrated in the plains along the east coast. The capital São Tomé is in the district of Água Grande. The climate is equatorial with average temperatures around 25–27 °C. Three seasons were categorized based on rainfall in this study (Source: World Weather Online- https://www.worldweatheronline.com/sao-tome-weather-averages/sao-tome/st.aspx), which are rainy season from January to May, dry season from June to September, and heavy rain season from October to December.

### Malaria vector control methods in STP

The time chart of malaria interventions from 2003 to 2016 was shown in Additional file 1: Fig. S1. Most interventions were introduced or scaled-up after 2004. In the early stage, STP applied three rounds of country-wide IRS with alpha-cypermethrin at a dosage of 50 mg/m^2^ from 2004 to 2007 with population coverage of 93.8% [19]. From 2007 to 2013, the programme shifted to using *Bti* for outdoor larval control. Two kinds of *Bti* formulation were applied in STP. Granular *Bti* 200 (VectoBac^®^ G, serotype H-14, Lot number 145-077-N8, 200 international toxic units (ITU)/mg, Valent Bioscience Corporation, Libertyville, USA) was used at the dosage of 1 g/m^2^. Each province hired three technicians who spread the granules outdoors every day, with one application per location per week. The formulation was temporarily changed to *Bti* 3000 (VectoBac^®^ WG, strain AM65-52 fermentation, Lot number 60215-08-03, 3000 ITU/mg, Valent Bioscience Corporation, Libertyville, USA) in São Tomé during 2011–2012, and a 10-L hand-press bucket pump was used to spray. The locations where *Bti* was applied were large areas of shallow accumulated water, permanent and temporary breeding sites.

### Mosquito collection

**Fig. 1:**
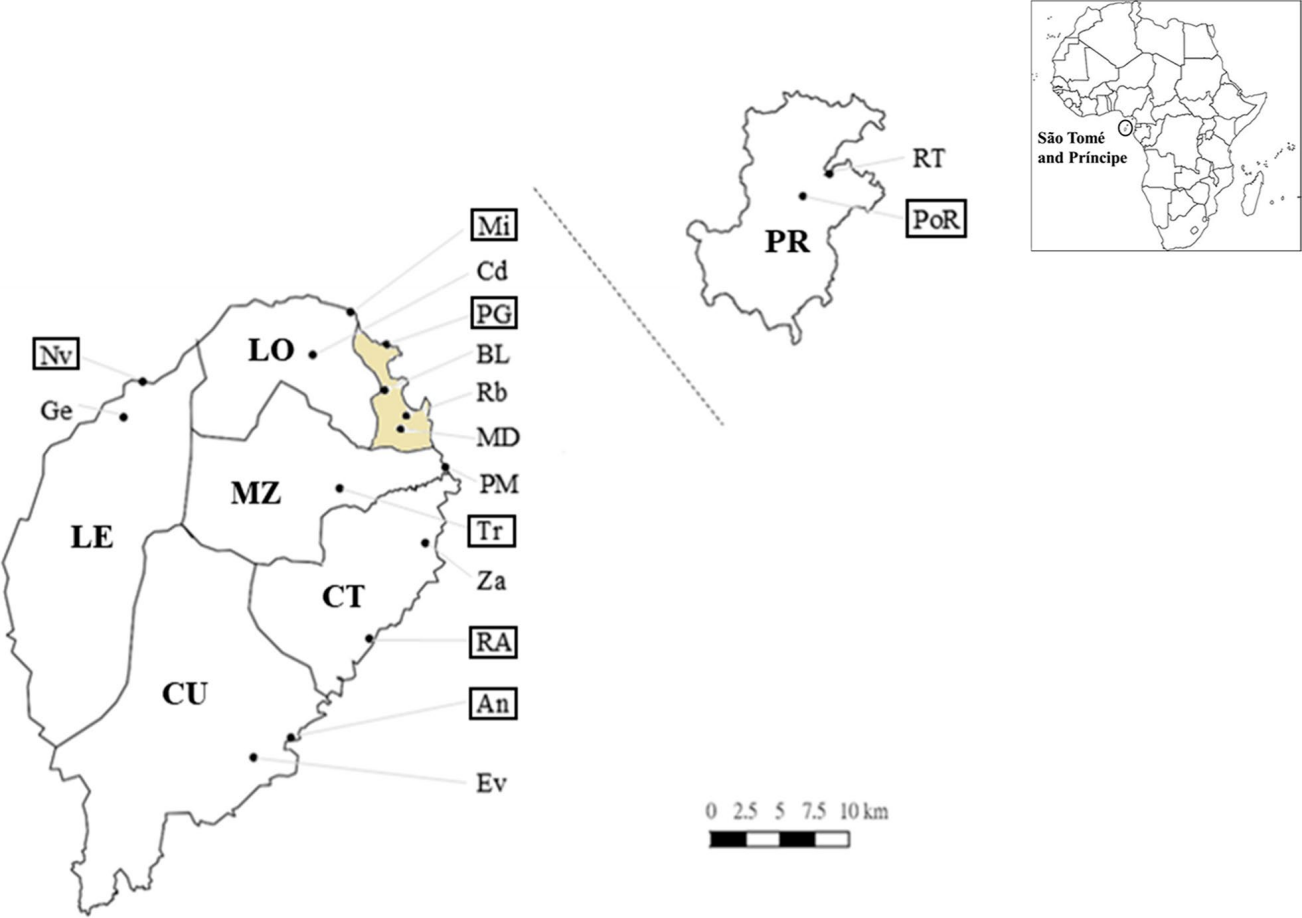
Map of mosquito collection sites in STP. Female adult mosquitoes were collected in two to four sites per district from 2010 to 2016. The seven districts are Príncipe (PR), Lobata (LO), Água Grande (AG, the colored region), Mé-Zóchi (MZ), Cantagalo (CT), Caué (CU), and Lembá (LE). The collection sites from north to south, east to west are Rua Trabalhadores (RT), Porto Real (PoR), Micolo (Mi), Conde (Cd), Praia Gamboa (PG), Bairro da Liberdade (BL), Riboque (Rb), Madre Deus (MD), Praia Melão (PM), Trindade (Tr), Zandrigo (Za), Ribeira Afonso (RA), Angolares (An), Emolve (Ev), Neves (Nv), and Generosa (Ge). The framed locations are the seven major sentinel sites which had integrated records of mosquito density. This map was drawn manually using QGIS ver 2.18

Female *Anopheles* mosquitoes were captured in a total of 16 sites from 2010 to 2016 (Fig. 1). Each district has two collection sites, except for Água Grande which has four collection sites. Mosquito samples were collected by human landing catches (HLCs) from 9 p.m. to 2 a.m. inside and outside the house during their blood feeding period, and mosquito light traps (MLTs) hanging indoor and outdoor overnight. The preventive rapid screening tests (SD BIOLINE Malaria Ag P.f/Pan test, Abbott Laboratories, Illinois, USA) and mefloquine prophylaxis (Apotex Inc., Ontario, Canada) [35, 36] were provided for diminishing malaria infection risks of local workers who have been trained and in charge of capturing mosquitoes. The procedure was approved by the Institutional Research Ethics Committee: NTUH-REC No.: 201110023RD. The collected mosquitoes were preserved in tubes with cotton flap and silica gel for laboratory analysis [37]. After morphological identification, samples were stored in room temperature for molecular analyses.

### Mosquito density calculation

Mosquito density was calculated in seven major sentinel sites (Fig. 1, framed locations) including Porto Real (PoR), Micolo (Mi), Praia Gamboa (PG), Trindade (Tr), Ribeira Afonso (RA), Angolares (An), and Neves (Nv). Density was calculated by numbers of human landing captured adult mosquitoes/house/working hour/month. Annual average mosquito density of indoor and outdoor HLCs from 2004 to 2016 was calculated and compared between Príncipe and São Tomé islands and different control strategies. In order to explore the detailed density trend during 2010 to 2016, monthly density were compared between different collection methods, districts, years, and seasons by one-way analysis of variance (ANOVA) and Tukey’s test using R version 3.5.1.

### Mosquito DNA extraction

Genomic DNA was extracted from 1923 individual mosquitoes collected from 2010 to 2016. The SpeedMill PLUS instrument (Analytik Jena AG, Jena, Germany) was used to homogenize mosquitoes, and the Genomic DNA Mini Kit for Tissue (Geneaid Biotech Ltd., Taipei, Taiwan) was used to extract DNA. Each mosquito was homogenized in a 1.5 mL Eppendorf tube containing a 3 mm sterilized stainless steel bead. A volume of 400 μL lysis buffer was added to break the mosquito debris. Samples were then incubated and washed according to the protocol from Genomic DNA Mini Kit for Tissue (Geneaid Biotech Ltd., Taipei, Taiwan). A volume of 50–100 μL DNA was eluted from each mosquito sample.

### Polymerase chain reaction (PCR) and sequencing

About 25–30 samples collected per year among districts were selected for *IGS* form-specific identification and *COI* sequencing (Additional file 2: Table S1). From 2010 to 2016, a total of 205 mosquitoes were pre-identified by form-specific *IGS* using PCR-restriction fragment length polymorphism (RFLP) method described by Fanello et al. [38]. Twenty PCR products of *IGS* were sequenced to confirm the form-specific RFLP results. Mitochondrial *COI* was amplified using three pairs of primers (Additional file 2: Table S2) for construction of a full length of 1506-bp sequence in the 205 samples [39, 40]. PCR analysis used 10 μL of 2X HotStarTaq Master Mix (Qiagen, Hilden, Germany), 10 μM of forward and reverse primers, 2 μL of DNA template, and 6 μL of RNAse free water to a total volume of 20 μL. After confirming PCR products by 2% agarose gel electrophoresis, the products were sequenced by Applied Biosystems 3730*xl* DNA Analyzer (Thermo Fisher Scientific, Waltham, USA). If any mixed nucleotide appeared in the direct sequence of PCR product, cloning system by PCR-4-TOPO and One Shot™ TOP10 Chemically Competent *Escherichia coli* (Thermo Fisher Invitrogen, Carlsbad, USA) would be performed. The plasmid of the colony would be sequenced using M13 primers to confirm the nucleotide polymorphism in the sequence.

### *COI* sequence analysis

The 205 *COI* sequences were aligned using Lasergene SeqMan ver 7.1 (DNASTAR Inc., Madison, USA) and BioEdit ver 7.0 [41]. Phylogenetic analysis was carried out by MEGA7 [42]. Neighbor-joining (NJ) tree of *COI* sequences was bootstrapped for 1000 times using Kimura 2-parameter model [43]. DNA polymorphism, Tajima’s D_T_, Fu’s Fs, Fu & Li’s F* and D* neutrality tests were performed by Dnasp6 [44]. A statistical parsimony TCS network was depicted using PopART ver 1.7 [45] to infer the genealogical relationships among haplotypes. Pairwise fixation index (F_ST_) and hierarchical analysis of molecular variance (AMOVA) were analysed by Arlequin ver 3.5 [46]. Gene flow (Nm, number of migrants) was calculated by (1 − F_ST_)/4*F_ST_ [47].

### Knockdown resistance genotyping and calculation of mutation frequency

The 1014F *kdr* mutation was screened in the DNA of 1923 mosquitoes by allele-specific PCR as described in Martinez-Torres et al. [48]. Three genotypes, susceptible homozygotes (SS), resistant homozygotes (RR), and heterozygotes (RS) were identified by gel electrophoresis after PCR. Four samples from each genotype were sequenced to confirm the results from allele-specific PCR. The N1575Y *kdr* mutation was also screened in 30 samples across the study period using PCR-sequencing [49]. Acetylcholinesterase-1 (*ace*-*1*) G119S target site mutation of carbamate and organophosphate was screened in 345 samples across the study period using PCR–RFLP method [50].

The *kdr* mutation frequency and 95% confidence interval (CI) were estimated by Genepop ver 4.2 using maximum likelihood estimation [51, 52]. Comparison of *kdr* mutation frequencies between different districts was assessed by one-way ANOVA and Tukey’s test. The *kdr* mutation frequency trend by year was assessed by Pearson correlation analysis.

### Risk characterization of geo-environmental and vectorial factors to malaria incidence

To explain malaria incidence with geo-environmental and vectorial factors in STP, a negative binomial regression model was fitted. Malaria cases were diagnosed and confirmed by microscopic examination of blood slide and rapid diagnostic test. The diagnostic results would be imported into the electronic medical record system. Malaria case numbers of aged < 5 and ≧ 5, and slide positivity rate (SPR) of fever patients in the medical record system were calculated and organized by the Taiwan Anti-Malaria Advisory Mission. Case management was carried out for 28 days post-treatment to confirm whether patients were completely cured. Population estimates by district per year were referred from official documents at Instituto Nacional de Estatística, São Tomé and Príncipe (https://www.ine.st/index.php/publicacao/documentos/category/76-dados-localidade-projecoes). This study assumed that the monthly population per district was the same as the annual estimates. Monthly incidence was calculated by number of monthly malaria cases by district/annual population estimates by district.

The considered covariates were (i) area divided by human population density and geography (urban: AG, MZ, LO, CT; rural: LE, CU; offshore island: PR) (ii) rainfall for 1-month lag (data retrieved from World Weather Online: https://www.worldweatheronline.com/sao-tome-weather-averages/sao-tome/st.aspx) (iii) entomological inoculation rate calculated by mosquito density of indoor or outdoor HLCs multiplied a sporozoite rate of 0.5% [3] (assuming that the sporozoite rate was fixed under low transmission scenario) (iv) *kdr* mutation frequency by season (because the sample size of analysed mosquitoes per month was too small, *kdr* mutation frequency was calculated per season). All statistical analyses were performed by R version 3.5.1.

## Results

### Dynamic changes of the density of *Anopheles* mosquitoes

**Fig. 2:**
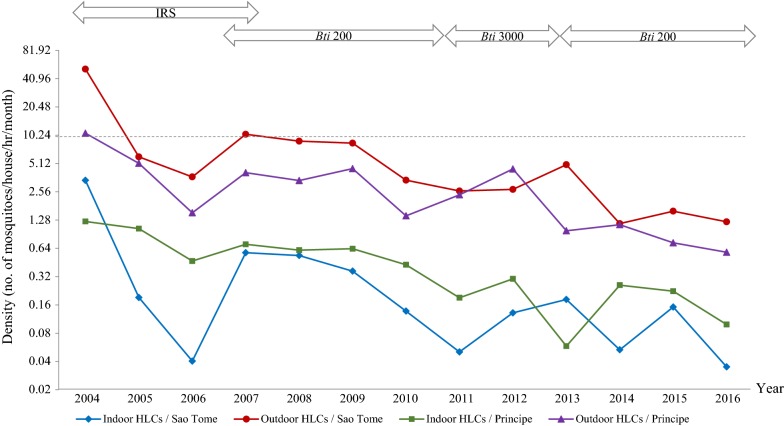
Annual average density of *Anopheles* mosquitoes by outdoor and indoor human landing catch (HLC) in São Tomé and Príncipe from 2004 to 2016. The average indoor and outdoor mosquito density in Sao Tome (blue and red line, respectively) was calculated from six sentinel sites in six districts (Micolo, Praia Gamboa, Trindade, Ribeira Afonso, Angolares, and Neves). The indoor and outdoor mosquito density in Príncipe (green and purple line, respectively) was calculated at one collection site, Porto Real. The Y axis (density) was shown in a scale factor of two. Mosquito density was substantially decreased during IRS control period (2004–2006). Larval control by *Bti* had long-term efficacy to keep the outdoor mosquito density under 10 (the dashed line)

The annual average of *Anopheles* mosquito density by indoor and outdoor HLCs from 2004 to 2016 under different control strategies was shown in Fig. 2. First, mosquitoes captured by outdoor HLCs (density range: 0.58–51.69) was higher than indoor HLCs (density range: 0.04–3.39). Second, mosquito density of outdoor HLCs in São Tomé (range: 1.17–51.69) was mostly higher than that in Príncipe (range: 0.58–10.75). However, mosquito density of indoor HLCs was mostly higher in Príncipe than São Tomé. Third, mosquito density on both islands were at the highest at the beginning of IRS application, which outdoor density in São Tomé and Príncipe were 51.69 and 10.75, and indoor density were 3.39 and 1.24, respectively. After the first round of IRS programme completed at the end of 2004, the outdoor density in the next year substantially dropped by 8.57-fold in São Tomé and 2.09-fold in Príncipe, and the indoor density dropped by 17.84-fold in São Tomé and 1.19-fold in Príncipe. This result demonstrated that IRS by alpha-cypermethrin could rapidly decrease *Anopheles* density at an early stage. However, after the second and third round of IRS completed in 2005 and 2006, the density slightly increased during 2006 to 2007. The programme, therefore, shifted focus to outdoor larval control using *Bti* 200 starting from 2007. During the period of *Bti* control, the density was gradually declined except for a fluctuation in 2012–2013 when STP temporarily changed from *Bti* 200 to *Bti* 3000. Nevertheless, outdoor larval control by *Bti* showed long-term efficacy for controlling mosquito density below 10.

**Fig. 3:**
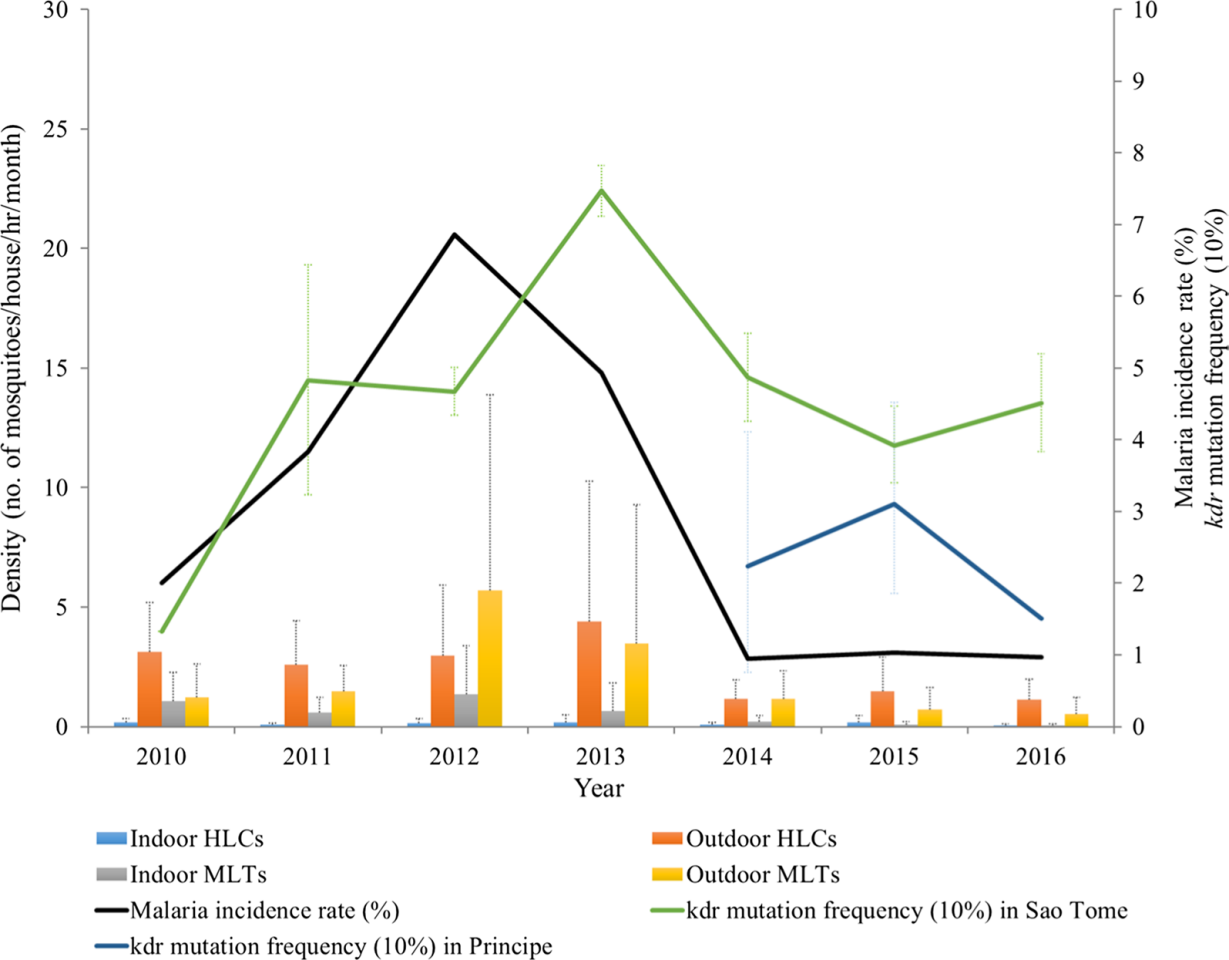
Malaria incidence rate, mosquito density, and *kdr* mutation frequency in STP from 2010 to 2016. The histogram shows average mosquito density by four collection methods (HLCs = human landing catches; MLTs = mosquito light traps), and the bars show the standard deviation. Black line is the malaria incidence rate (case numbers/total population*100%). Green and blue lines are the *kdr* mutation frequency (10% per unit) in São Tomé (green) and Príncipe (blue). The bars on the estimated *kdr* mutation frequency are 95% confidence intervals

Monthly vector density from 2010 to 2016 in seven sentinel sites showed the mean density of outdoor HLCs and MLTs (1.87 and 1.78) were significantly higher than those by indoor HLCs and MLTs (0.13 and 0.55). Concluding density from four methods of mosquito collection, spatial variations showed urban districts, Água Grande (AG, mean = 2.18) and Lobata (LO, mean = 2.01), had significantly higher vector density compared to rural districts, Lembá (LE, mean = 0.49) and Caué (CU, mean = 0.46). Temporal comparison showed a significant lower density in 2014–2016 (mean = 0.34–0.56) compared to 2010–2013 (mean = 1.30–2.49). The lower mosquito density occurred during 2014 to 2016 (annual average = 1.17–1.59) was in accordance with the low malaria incidence rate (0.9–1%) at the same period (Fig. 3). The seasonal effect of mosquito density was shown in Additional file 2: Table S3. Mean density was significantly lower in dry season compared to heavy rain season (mean difference = − 0.64, p < 0.001) and rainy season (mean difference = − 0.67, p < 0.001). No significant difference was observed between heavy rain season and rainy season.

### Sequence analysis of malaria vector

*IGS* molecular M form was confirmed in a total of 205 mosquitoes using PCR–RFLP (GenBank accession no. KT284724). There was only 1 *IGS* haplotype (350 bp) in STP. The *IGS* sequence revealed polymorphisms compared to *An. gambiae* in Benin (GenBank accession nos. AF470112–470116) with the identity of 98.57–99.71%.

There were 48 haplotypes (GenBank accession nos. MH025842–025880 and MK330882–330890) found in 205 *COI* sequences (Additional file 2: Table S1). The 205 *COI* sequences of *An. coluzzii* included 39 singleton variable sites and 11 parsimony informative sites. Estimated transition over transversion bias was 9.41, resulting in 45 synonymous changes, four amino acid changes, and one ambiguous amino acid.

**Fig. 4:**
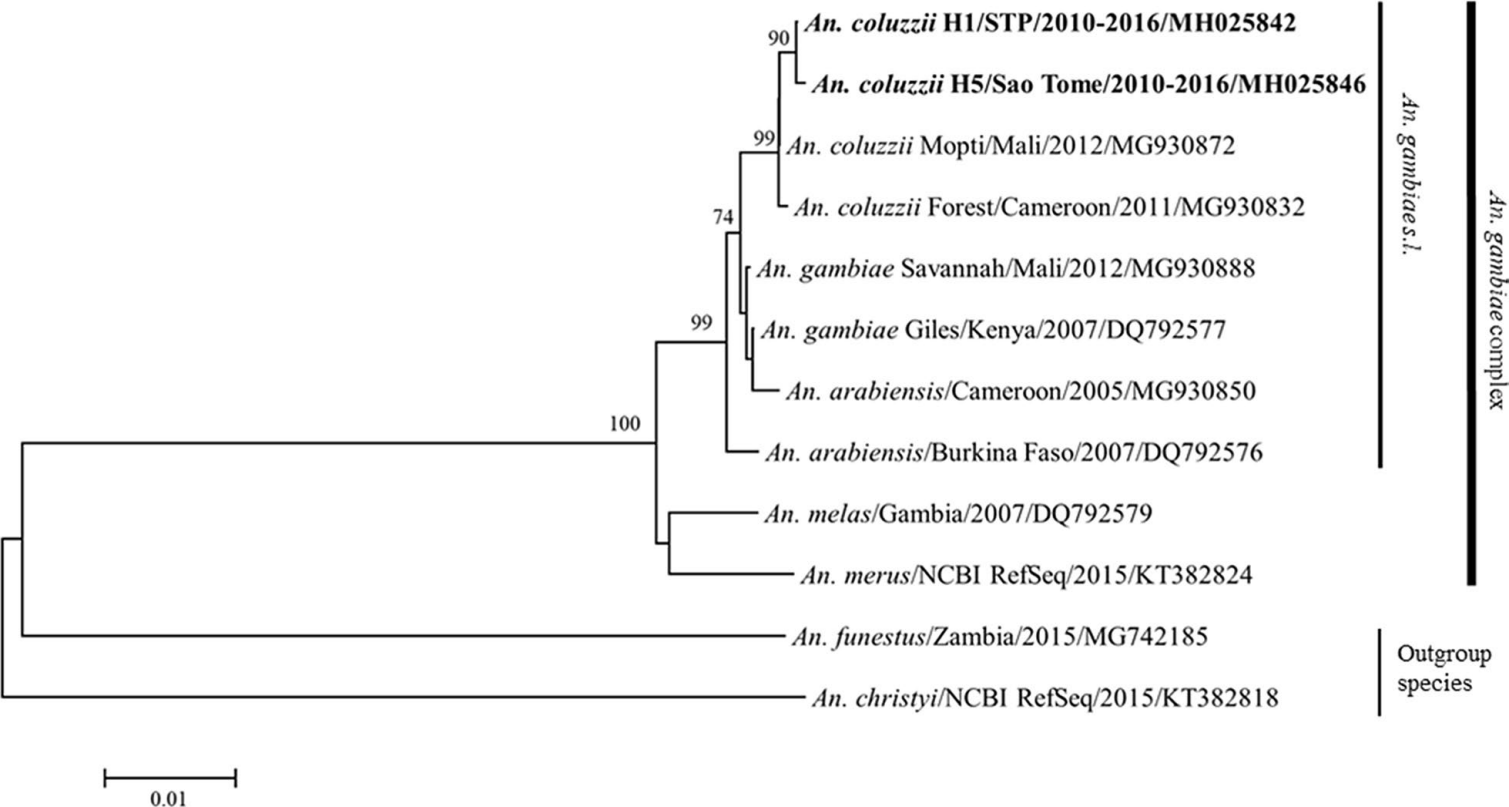
Phylogenetic tree constructed by *COI* sequences (1506 bp) of African *Anopheles* species. Sequences are denoted by Species/Origin/Year/Accession number. NCBI RefSeq means the sequence is originated from the NCBI Reference Sequence Genome Project. Two major haplotypes (H1 and H5) in STP are shown in bold. The number on the branch is the bootstrap value with 1000 replicates. Bootstrap value which is under 70 is not shown in this figure

**Fig. 5:**
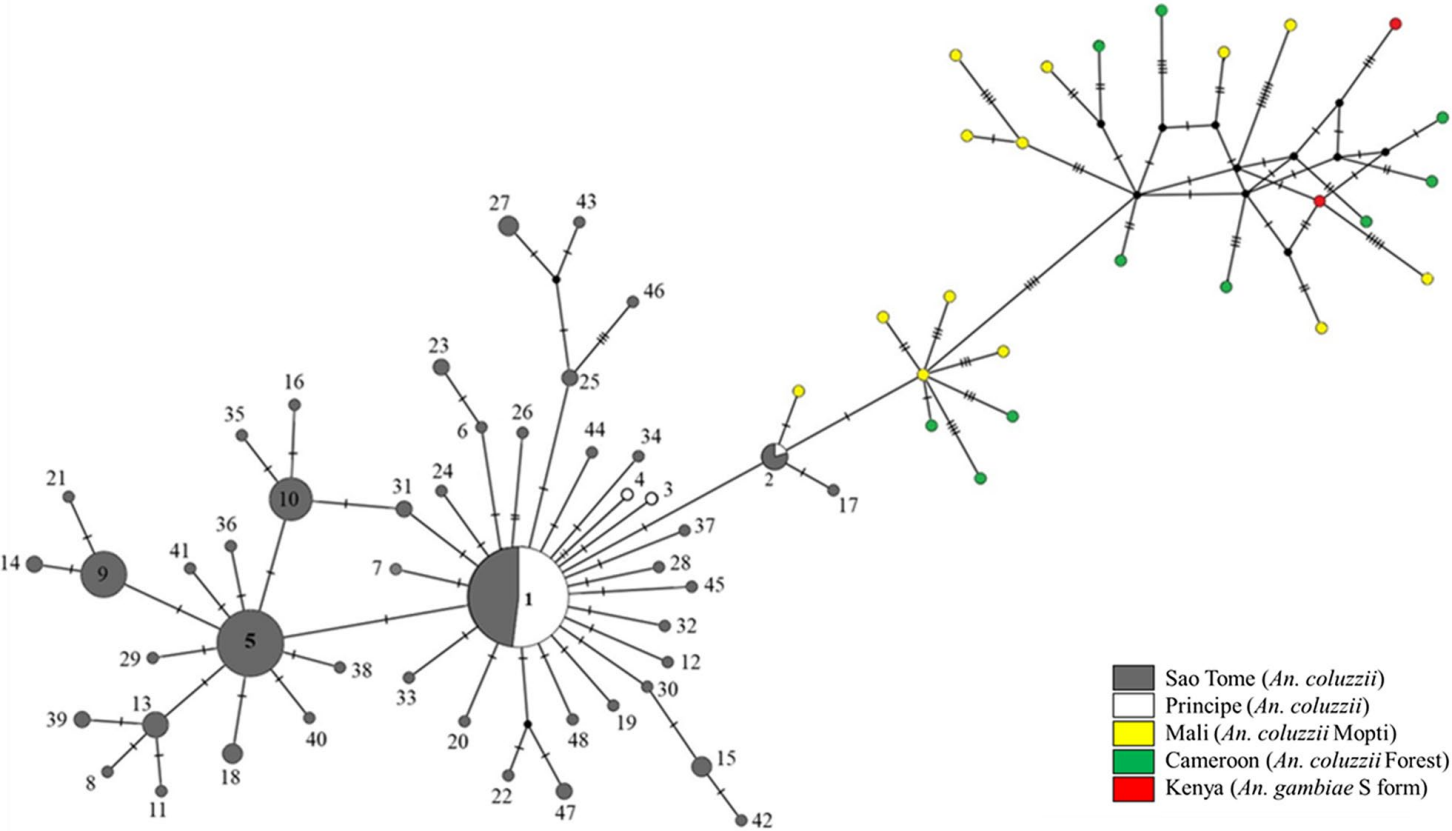
TCS haplotype network of *COI* sequences (1506 bp). A total of 48 haplotypes in São Tomé and Príncipe are displayed in circles with gray and white color, respectively. The size of the circle is proportional to its frequency. One node indicates one nucleotide difference. Vector population in Príncipe harbors only four haplotypes which H1 is the dominant (93%, 40/43), while population in São Tomé owns much diverse haplotypes. Other colors are reference sequences of *An. gambiae s.s.* from West, Central, and East African countries

Haplotype 1 and 5 (H1, H5) were the two major *COI* haplotypes in STP (Additional file 2: Table S1). H1 was dominant in Príncipe population (93%, 40 sequences out of 43), whereas H5 was only found in São Tomé population (21.6%, 35/162). The phylogenetic tree was constructed by NJ analysis (Fig. 4) enrolling the two major *COI* haplotypes in STP with other reference sequences including *An. gambiae* sibling species, *An. funestus* (another major malaria vector complex in Africa), and *Anopheles christyi* (non-malaria vector but closely related to *An. gambiae* complex). Gene genealogies was depicted by TCS network within *An. gambiae* sensu stricto (*s.s.*) in Fig. 5. Both phylogenetic tree and TCS network showed that *An. coluzzii* in STP formed a subgroup with the most similarity to the *An. coluzzii* in West and Central African country including Mali and Cameroon.

Tajima’s D_T_, Fu’s Fs, Fu & Li’s F* and D* neutrality tests all showed significant negative values, indicating an excess of rare alleles in *COI*, and suggesting either a recent selective sweep or population expansion (Table 1). *Anopheles coluzzii* on Príncipe offshore island displayed simplex genetic structure with very low haplotype and nucleotide diversity. AMOVA and pairwise differentiation index showed that the Príncipe population had significant genetic differentiation (mean F_ST_ = 0.16, p < 0.001) with the population from São Tomé (Table 2 and Additional file 2: Table S4) which may be due to geographic isolation. Although diverse *COI* haplotypes were identified in São Tomé, pairwise F_ST_ showed no significant differentiation between districts in São Tomé except for rural areas- Lembá and Caué.

**Table 1:** DNA polymorphism and neutrality tests of *COI* in *An. coluzzii* from STP

Island	No.	H (%)	S	K	Hd (SD)	π (SD)	D_T_	Fs	D*	F*
São Tomé	162	46 (28.4)	48	1.953	0.887 (0.015)	0.0013 (0.00008)	− 2.324**	− 56.027**	− 5.962**	− 5.142**
Príncipe	43	4 (9.3)	4	0.186	0.136 (0.071)	0.00012 (0.00007)	− 1.875*	− 3.283**	− 3.289*	− 3.136**

*No.* number of sequences, *H (%)* number of haplotypes (H/No.*100%), *S* number of segregating sites, *K* average number of nucleotide differences, *Hd* haplotype diversity, *SD* standard deviation, *π* nucleotide diversity; D_T_ = Tajima’s D; Fs = Fu’s Fs, D* = Fu & Li’s D* test; F* = Fu & Li’s F* test; *p < 0.05; **p < 0.02. The statistics are performed by Dnasp6

**Table 2:**
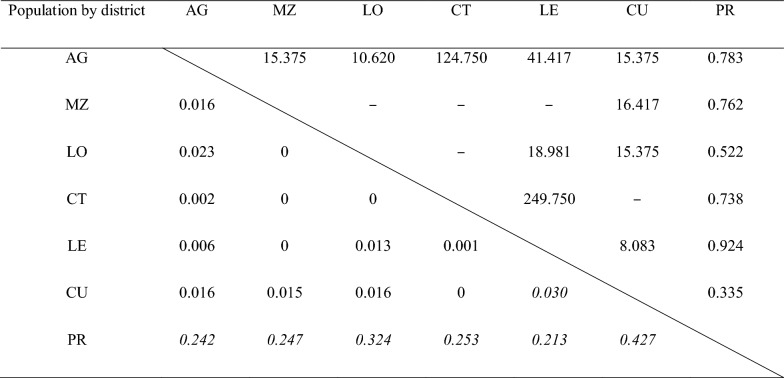
Pairwise differentiation index (F_ST_) and gene flow (Nm) of *An. coluzzii* population between districts of STP

Below diagonal is the differentiation index (F_ST_) and above is the gene flow (Nm) between populations. Nm is estimated by (1 − F_ST_)/4*F_ST_. F_ST_ values in italic are significant (p < 0.05). Endash (–) means the two populations have infinite gene flow. F_ST_ between population of Príncipe (PR) and districts in São Tomé are high and significant. Population differentiation between districts in São Tomé are low and not significant except for Lembá and Caué. Correspondingly, gene flow of the Príncipe population is the most limited (Nm < 1). Statistics are performed by Arlequin ver 3

### Spatial and temporal variations of *kdr* mutation

The *kdr* L1014F mutation was confirmed (GenBank accession nos. KT284726–284728), but no L1014S mutation was detected in *An. coluzzii* from STP. The *kdr* 1014F allele frequency by district per year was shown in Additional file 2: Table S5. The means of *kdr* mutation frequencies of *An. coluzzii* were significantly different between the two islands (F = 9.31, p = 0.003) in which São Tomé (mean frequency = 44.77%) was higher compared to Príncipe (mean frequency = 15.82%). Within São Tomé island, means of *kdr* mutation frequencies were not significantly different according to one-way ANOVA test (F = 1.43, p = 0.23) among vector mosquitoes collected from six districts.

Annual trend of *kdr* mutation frequency from 2010 to 2016 was shown in Fig. 3. The homozygous resistant allele (RR) was only detected in one mosquito sample (1.75%, 1/57) in 2010, indicating that *kdr* L1014F mutation was not widely distributed in the vector population at that time. However, the total *kdr* mutation frequency in São Tomé had gradually increased, reaching highest frequency in 2013 (Pearson’s r = 0.80, p < 0.001), 1 year after the malaria outbreak in 2012, but significantly decreased afterwards (Pearson’s r = − 0.49, p < 0.001). Four collection sites from Príncipe, Lobata, and Caué (Porto Real, Conde, Angolares, Emolve) had no resistant homozygotes (RR) detected at the end of 2016. The total *kdr* mutation frequency in Príncipe was relatively low (0–31%) except for a slight increase from 2014 to 2015.

In addition, subsets of samples were selected from each collection site and year to detect other target site mutations. Results showed that no *kdr* N1575Y (0%, 0/30) and *ace*-*1* G119S (0%, 0/345) mutations were detected throughout the whole study period.

### Transmission risks of environmental and vectorial factors to the malaria incidence

**Fig. 6:**
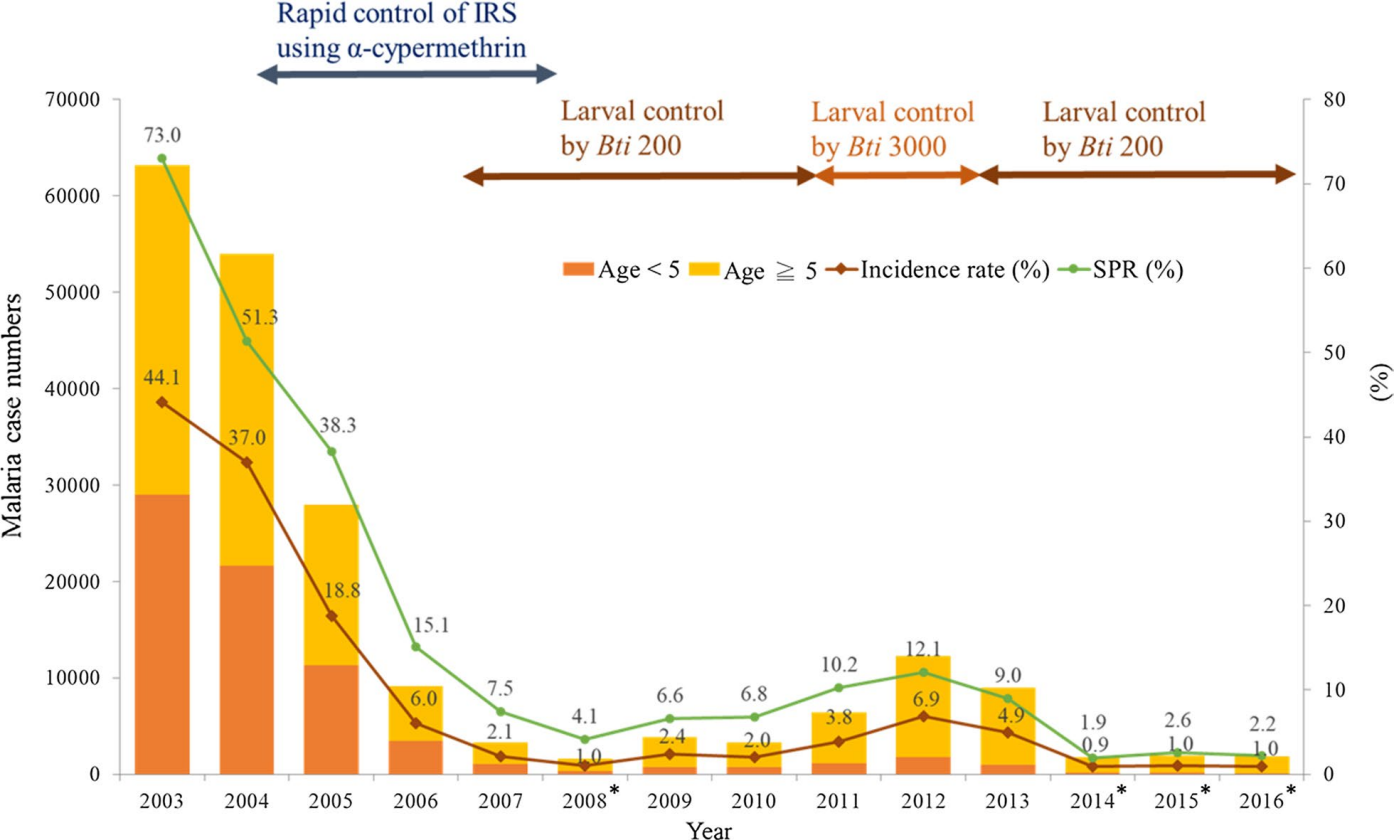
National malaria case numbers, incidence rate, and slide positivity rate (SPR) in STP from 2003 to 2016. *The year that reaches pre-elimination criteria: SPR < 5% (referred from WHO [53]), annual incidence rate ≦ 1%, and case numbers < 2000

The national malaria case numbers, incidence rate, and SPR from 2003 to 2016 in STP was shown in Fig. 6. Malaria case numbers and incidence rate had substantially decreased by 16.3 and 17.6-fold during IRS control period (2004–2007), respectively, and kept under incidence rate of 5% after shifting to *Bti* control since 2007, except for a small rise in 2012 (incidence rate = 6.9%). The recent incidence rate after 2014 persisted at the lowest point (0.9–1%). The case proportion of the high-risk group, children under age of five, have gradually decreased from 45.87% in 2003 to 5.80% in 2016. On the contrary, 75–95% of the malaria cases were found in age ≧ 5 since 2008. National SPR showed corresponding trend with incidence rate (correlation coefficient = 0.987), and reached under 5% (WHO pre-elimination criterion [53]) in 2008, 2014, 2015, and 2016 when the annual incidence rate was around 1% and the case number was under 2000.

Malaria case numbers and incidence rate in Príncipe offshore island was decreased by 13.5 and 14.2-fold, respectively, during IRS control period from 2004 to 2007 (Additional file 1: Fig. S2). After shifting IRS to larval control by *Bti* 200 from 2007, Príncipe have been showing lower case numbers (2–51 cases per year) and incidence rate (under 1%) since 2008, and reached less than one case per 1000 person (< 0.1%) since 2014. This result indicated that Príncipe island was on the progress from malaria pre-elimination to elimination phase (criterion: < 1 case/1000 population at risk) [51].

To link the geo-environmental data with local malaria incidence, human population density by area and rainfall were enrolled as important factors that could affect malaria transmission. The census by district was conducted in 2012 (Additional file 2: Table S6). Over 80% of population are concentrated in the eastern part of São Tomé (AG, MZ, LO, and CT). In mountainous regions LE and CU resided 8.2% and 3.4% of total population, respectively. Príncipe resided 4.1% of total nationals. The seasonality of malaria incidence could be affected by rainfall with a time lag of 1 month (Additional file 1: Fig. S3). Also, when the monthly incidence rate was higher than 0.25% (25 cases per 10,000 persons) at the end of the year, the epidemic could be extended to the upcoming year.

From Fig. 3, a fluctuation between mosquito density, *kdr* mutation frequency and malaria incidence was observed during 2010 to 2016. To further understand the impacts of these vector characteristics and geo-environmental factors to the malaria incidence, a negative binomial regression model was constructed. The response variable was monthly malaria incidence rate in the seven districts during 7 years, and therefore the number of observations was 12 × 7 × 7 = 588. Details of the covariates were described in Table 3. This best model was selected by the lowest Akaike information criterion (AIC) value (2949.6). Malaria incidence in urban and rural area were 11.50 (95% CI 5.54–23.89) and 9.33 (95% CI 4.44–19.58) fold higher than Príncipe, respectively. The incidence of previous month would affect the latter by a risk ratio of 16.39 (95% CI 12.17–22.07). Incidence could increase by 1.25-fold (95% CI 1.13–1.39) when the rainfall in previous month increased 100 mm. Entomological inoculation rate (EIR, %) which was transformed from mosquito density was not significant in indoor EIR, but significant in outdoor EIR which could increase the incidence by 1.07-fold (95% CI 1.04–1.11). On the other hand, incidence could increase by 1.06-fold (95% CI 1.02–1.10) when *kdr* mutation frequency was increased by 10%. In conclusion, the geo-environmental factors including area and rainfall, and mosquito characteristics including outdoor EIR (density) and *kdr* mutation were significant factors that could increase malaria infection risks in STP.

**Table 3:** Results of negative binomial regression model (no. of observations = 588)

Covariates	Estimate	Std. error	*p* value	Risk ratio (95% CI)
Urban area^a^ (AG, MZ, LO, CT)	2.443	0.373	*< 0.001*	11.50 (5.54–23.89)
Rural area^a^ (LE, CU)	2.233	0.378	*< 0.001*	9.33 (4.44–19.58)
Incidence rate (%) in previous month	2.796	0.152	*< 0.001*	16.39 (12.17–22.07)
Rainfall amount (100 mm) in previous month	0.225	0.052	*< 0.001*	1.25 (1.13–1.39)
Indoor EIR^b^ (%)	− 0.217	0.194	0.263	0.80 (0.55–1.18)
Outdoor EIR^c^ (%)	0.072	0.016	*< 0.001*	1.07 (1.04–1.11)
*Kdr* mutation frequency^d^ (10%)	0.060	0.019	*0.0017*	1.06 (1.02–1.10)

The response variable is monthly incidence rate in 7 districts from 2010 to 2016

^a^Reference area is the offshore island-Príncipe

^b^Indoor EIR (%) is calculated by monthly mosquito density of indoor HLCs multiplied sporozoite rate (0.5%) which is referred from Pinto et al. [4]

^c^Outdoor EIR (%) is calculated by monthly mosquito density of outdoor HLCs multiplied sporozoite rate (0.5%) which is referred from Pinto et al. [4]

^d^*Kdr* mutation frequency is calculated per season using Genepop ver 4.2. *Kdr* mutation frequency is assumed the same per month in the same season. The risk ratio is modelled by an increase of 10% in *kdr* mutation frequency

## Discussion

This longitudinal study described malaria vector characteristics in STP including density changes, population composition, insecticide resistance status, and the implications for malaria epidemics. Both IRS and outdoor larval control by *Bti* had decreased vector density and malaria incidence of which the former showed short-term but rapid controlling effect, and the latter showed long-term and sustainable efficacy. The population composition in vector mosquitoes from Príncipe and São Tomé islands were different of which the former showed lower genetic diversity and *kdr* mutation compared to the latter. Outdoor EIR transformed from mosquito density, and *kdr* mutation were significant factors which could increase malaria incidence by 1.06–1.07 of risk ratio. Therefore, eliminating outdoor resistant mosquitoes should be of great priority to reduce malaria transmission in STP. This study also used the epidemiological data to better define the pre-elimination criteria for STP: (a) annual SPR < 5% and incidence rate ≦ 1% (b) annual case numbers < 2000 (c) annual average outdoor HLCs density < 1.6 mosquitoes/house/hr/month.

The simplex genetic structure and fewer *kdr* mutation in vector population from Príncipe are likely consequences of geographic isolation and absence of strong selection by insecticide due to very few malaria cases on this island (2–42 cases per year from 2010 to 2016). Only a small proportion of mosquitoes were detected harboring *kdr* mutant allele (2.3–9.6% RR, and 12.5–20.5% RS) in Príncipe from 2014 to 2015. On the contrary, São Tomé as the main island with more frequent transportation, malaria transmission and interventions [23], the vector population showed a more diverse genetic background and higher selection pressure by insecticide compared to Príncipe. However, due to the small area of São Tomé island (859 km^2^), less extent of difference was observed among districts in São Tomé for either *COI* or *kdr* markers. Only Lembá and Caué which were slightly isolated by surrounding mountain areas showed little but significant differentiation in *COI*. The heterogeneity and isolated characteristics of vector population between the two islands, as well as islands with the continental Africa were also supported by previous studies using mitochondrial, ribosomal, and microsatellite DNA markers of *An. gambiae* [4, 5, 54]. Marshall et al. analysed *ND5* and *ITS* sequences of *An. gambiae s.s.* and reported that the vector population in STP was possibly derived from West and Central African countries following human migration [5]. This could support our results that *COI* sequences of *An. coluzzii* in STP showed the most similarity to *An. coluzzii* from West and Central African countries. Although the nucleotide variations in *COI* was not high especially within *An. gambiae s.l.* [55], vector population in STP still formed its own subgroup in the phylogenetic analysis. Haplotype 1 which was the representative *COI* haplotype in Príncipe distributed on both islands across the whole sampling period. A single nucleotide polymorphism (SNP) in the loci of A1263G (H5) only appeared in samples from São Tomé but not those from Príncipe. This SNP was less seen but appeared in the *COI* sequences from *An. gambiae* in Cameroon [55], and Nigeria [40].

When examining the malaria epidemics under different vector control strategies, it is clear to see that IRS had short-term and rapid effectiveness in reducing both vector density and malaria epidemics. However, vector density was not well-controlled in the late stage of IRS application possibly due to the decreased susceptibility of insecticide in mosquitoes. Therefore, the alternative vector control method using *Bti* was then implemented at outdoor mosquito breeding sites in order to control mosquito larvae. Long-term application of *Bti* kept the outdoor vector density under a controlled level (under10 mosquitoes/house/hr/month), and successfully prevented malaria outbreaks after 2008, except for a fluctuation in 2012. This fluctuation may be due to the failure of shifting from *Bti* 200 to *Bti* 3000 in São Tomé for a period of time which decreased work efficiency since *Bti* 3000 application required workers to carry heavy spraying equipment. On the other hand, though the programme no longer emphasized IRS, in response to the WHO global malaria elimination policy, São Tomé continued to implement national or small-scale IRS with alpha-cypermethrin funded by Global Fund during 2009 to 2012, resulting in the detection of *kdr* mutation in our sampling period from 2010 to 2016. On the contrary, IRS was not accelerated in Príncipe where malaria incidence rate was under 1%. This may lead to a slightly higher indoor mosquito density observed in Príncipe than São Tomé, and a lower detection rate of *kdr* mutation in mosquitoes from Príncipe.

Since the malaria vector in STP is single species with limited gene flow, the *kdr* 1014F mutation may likely arisen independently due to the local insecticide selection. The mean of *kdr* 1014F mutation frequency in STP from 2010 to 2016 was 43.8% (annual range: 13.3–74.8%). This was relatively low compared to its neighboring country, the coastal area of Cameroon, which the *kd*r 1014F mutation frequency in *An. coluzzii* was 70–93% in 2015 [56]. *Kdr* mutation had a clear temporal trend which increased to the highest in 2013 and dropped afterwards in São Tomé. The anterior increase in *kdr* mutation may be contributed by the accelerated insecticide interventions during 2010 to 2013. Supportive data from WHO malaria reports showed the estimated IRS coverage in STP elevated from 40% in 2010 [57] to 84% in 2013 [58]. The modelled % of population with access to ITNs increased from 39% in 2010 [57] to 53% in 2013 [58]. After the breakpoint of 2013, *kdr* mutation had decreased and remained its frequency around 40%. This study suggested two possible explanations. First, it was correlated with the declining malaria incidence and IRS coverage during 2014 to 2016 [59]. Second, a new insecticide, Ficam was introduced for IRS usage in replacement of alpha-cypermethrin at the end of 2013 [60]. Ficam is a carbamate insecticide which has different mode of action to pyrethroids. The *ace*-*1* G119S is the known target site mutation against carbamate and organophosphate insecticides in *An. gambiae* [61]. However, 345 samples were screened for *ace*-*1* G119S and no mutation was detected by 2016, suggesting the vector may remain susceptible to carbamates. Another recent marker of target site mutation for pyrethroid is N1575Y which confers a significant additive benefit to L1014F [49]. However, 30 samples from 3 *kdr* genotypes (SS, RR, and RS) were pre-screened for this locus and none was detected mutant.

Early investigation reported the exophagy and exophily of malaria vectors in the STP [62, 63]. After long-term vector control interventions, *An. coluzzii* remained exophilic by evidence of more outdoor captures than indoors in our study. Entomological inoculation rate is an indicator of human exposure to infectious mosquitoes [64], and is determined by human biting rate which can be estimated using HLCs method [65], and sporozoite rate in vector mosquitoes. Previous entomological survey in STP showed the geometric mean of vector density was 0.5–44.3 per man per hour by outdoor HLCs, and a low sporozoite rate (0.3–0.6%) [3]. In this study, we used the arithmetic mean to calculate the average density. The monthly average mosquito density in outdoor HLCs ranged from 0 to 46 which was similar to previous findings (0.5–44.3) [3]. Since STP was under low malaria transmission scenario, we assumed that the sporozoite rate would remain low and less variable. Therefore, sporozoite rate of 0.5% referred from the aforementioned entomological survey was used for calculating EIR in our study [3]. The indoor EIR was not a significant risk factor in the model possibly due to the chemical and physical protections by IRS and ITNs. In contrast, the outdoor EIR was a significant risk factor owing to the exophagy of the local vector and the lack of protection in human hosts during outdoor activities.

The *kdr* L1014F amino acid substitution could alter the affinity of pyrethroids and DDT to bind on the sodium channel, resulting in the knockdown resistance [48]. A recent study conducted in Gambia examined the risk for *kdr* mutation but failed to provide conclusive findings due to the co-occurrence of *An. gambiae s.s.* and homozygous L1014F mutation [66]. In this study, *kdr* mutation was a significant risk factor to malaria incidence at a risk ratio of 1.06 (95% CI 1.02–1.10) for a 10% increase. The risk ratio was not high but similar to the results from the aforementioned study in Gambia where the odds ratio for a 10% increase in *kdr* mutation was 1.01–1.02 [66].

Malaria transmission in STP has strong seasonal effects mainly affected by rainfall. According to Teklehaimano’s report [67], malaria cases could appear 4–5 weeks following rainfall under hot environment (30 °C) when considering the lead time of mosquito life cycle (10 days), sporogony cycle (6 days), and incubation period (10–16 days) in human hosts. The transmission pattern in STP showed the peak incidence appeared 1 month after the end of rainy season. Therefore, this study used 1 month lag of rainfall to become the predictive value of malaria incidence. The temperature factor was not considered in this study due to low variability in STP. Human population density also have an impact on malaria transmission. Although several reports showed that area with higher population density has less malaria cases due to reduction of vector breeding sites and low EIR by urbanization [68, 69], it is not the case in STP since the highest malaria cases are found in the most densely populated district, Água Grande, and its neighboring districts Mé-Zóchi and Lobata are often close seconds. Lee et al. [23] pointed out the malaria outbreaks in STP may follow the main roads, suggesting that population distribution and migration play roles in the occurrence and spread of malaria epidemics. Knowing that the hot spot of malaria in STP is at the north-eastern part of São Tomé, malaria control strategies should be strengthened in this area.

Overall, this study exhibits the unique characteristics of vector population and malaria transmission under the island scenario. Geographic barriers by oceans, lakes, or mountains may result in limited gene flow among vectors and pathogens. Evidences from Bioko island, the nearby island to the STP, demonstrated the distance-determined genetic differentiation, exophagy and *kdr* 1014F mutation in *An. coluzzii* after long-term interventions [70] which were similar to the present findings in STP. Differentiation of vector population by mitochondrial DNA marker in the large lake area have also been found in the lacustrine islands located in the Lake Victoria which borders Kenya, Uganda and Tanzania [71]. Due to the small area and the relative simplicity of human mobility and vectorial-parasitological system on islands, it is much easier to eliminate malaria compared to the continental areas. Examples from East African islands such as the Comoros [72] and Zanzibar [73] showed opportunities to achieve malaria elimination by ACT administration and vector control interventions. Cape Verde, an archipelago of ten islands in West Africa, also achieved malaria pre-elimination according to the surveillance data from 2010 to 2016 [74]. However, imported infections from Africa continent, fluctuations of vector population, and sustainability of controlling interventions makes it challenging to ultimately eliminate or prevent recolonization of malaria on these islands [31]. It is essential to routinely monitor spatial–temporal vector dynamics, insecticide resistance in vectors, drug sensitivity in parasites, case detection and management, and to promote the health education in local residents, which all require financial and governmental support in order to maintain malaria control efficacy across these islands.

Although in parts of this study, secondary data or online resources were used due to the limited accessibility of direct data, this study still successfully explicated how vectorial and environmental factors would impact on the malaria epidemics in STP. During this longitudinal study, Taiwanese research team and Centro National de Endemias of STP collaborated to supervise and conduct routine inspections on the operation to ensure the accuracy and completeness of strategies implementation and data collection. Findings in this study focused on the geo-environmental and vectorial factors on malaria transmission. Future studies would be carried out to further investigate parasitological factors that could affect malaria control efficacy in STP.

## Conclusions

Different vector characteristics were found between São Tomé and Príncipe islands. *Anopheles* mosquito population in São Tomé showed higher density, genetic diversity in *COI*, and *kdr* mutation frequency compared to the population in Príncipe. Temporal trend showed mosquito density and *kdr* mutation elevated to the highest followed by the outbreak during 2012 to 2013 period. Geo-environmental factors including area and rainfall, and vectorial factors including outdoor EIR transformed from density and *kdr* mutation were factors that could significantly affect local malaria transmission.

Vector control by IRS could rapidly decrease *Anopheles* density and malaria epidemics in STP. Outdoor larval control using *Bti* is a sustainable long-term approach for controlling local *An. coluzzii* with exophilic feature and insecticide resistance problem.

To pre-eliminate malaria in STP, the following criteria should be sustained: (a) annual SPR < 5% and incidence rate ≦ 1% (b) annual case numbers < 2000 (c) annual average outdoor HLCs density < 1.6 mosquitoes/house/hr/month. Malaria prevention by vector control strategies should be intensified at urban districts during the epidemic season from May to June in order to successfully reduce malaria disease burden in this island nation.

## Supplementary information

**Additional file 1: Fig. S1.** Time chart of malaria interventions in STP. **Fig. S2.** Malaria case numbers and incidence rate in Príncipe from 2003 to 2016. **Fig. S3.** Monthly rainfall and malaria incidence rate in STP from 2010 to 2016.

**Additional file 2: Table S1.** *COI* haplotypes of *An. coluzzii* in STP. **Table S2.** Primers for *COI* PCR. **Table S3.** Comparisons of mosquito density in different seasons. **Table S4.** AMOVA analysis of genetic variations in *An. coluzzii* populations by *COI*. **Table S5.** Frequency of *kdr* L1014F mutation in 1,923 *An. coluzzii* from 7 districts during 2010 to 2016. **Table S6.** Nationwide population census in 2012 in STP.

## Data Availability

All data generated or analysed during this study are included in this published article and its additional files.

## Abbreviations

STP: São Tomé and Príncipe
IRS: indoor residual spraying
*Bti*: *Bacillus thuringiensis israelensis*
*COI*: cytochrome *c* oxidase subunit I
*kdr*: knockdown resistance
EIR: entomological inoculation rate
*IGS*: intergenic spacer
IVM: integrated vector management
LLINs: long-lasting insecticidal nets
WHO: World Health Organization
ITNs: insecticide-treated nets
AG: Água Grande
MZ: Mé-Zóchi
LO: Lobata
CT: Cantagalo
LE: Lembá
CU: Caué
PR: Príncipe
ITU: international toxic units
HLCs: human landing catches
MLTs: mosquito light traps
ANOVA: analysis of variance
PCR: polymerase chain reaction
RFLP: restriction fragment length polymorphism
NJ: Neighbor-joining
F_ST_: pairwise fixation index
AMOVA: analysis of molecular variance
Nm: number of migrants
SS: *kdr* susceptible homozygotes (1014 L/L)
RR: *kdr* resistant homozygotes (1014 F/F)
RS: *kdr* heterozygotes (1014 L/F)
*Ace*-*1*: acetylcholinesterase-1
CI: confidence interval
SPR: slide positivity rate
