# Supplementary material for: Effects of indoor residual spraying and outdoor larval control on Anopheles coluzzii from São Tomé and Príncipe, two islands with pre-eliminated malaria
Source: Malar J. 2019 Dec 5;18:405. doi: 10.1186/s12936-019-3037-y (PMC6896513; doi:10.1186/s12936-019-3037-y)
Supplement: Supplementary file 1 — Additional file 1: Fig. S1. Time chart of malaria interventions in STP. Fig. S2. Malaria case numbers and incidence rate in Príncipe from 2003 to 2016. Fig. S3. Monthly rainfall and malaria incidence rate in STP from 2010 to 2016. [file 12936_2019_3037_MOESM1_ESM.pptx]

## Slide 1
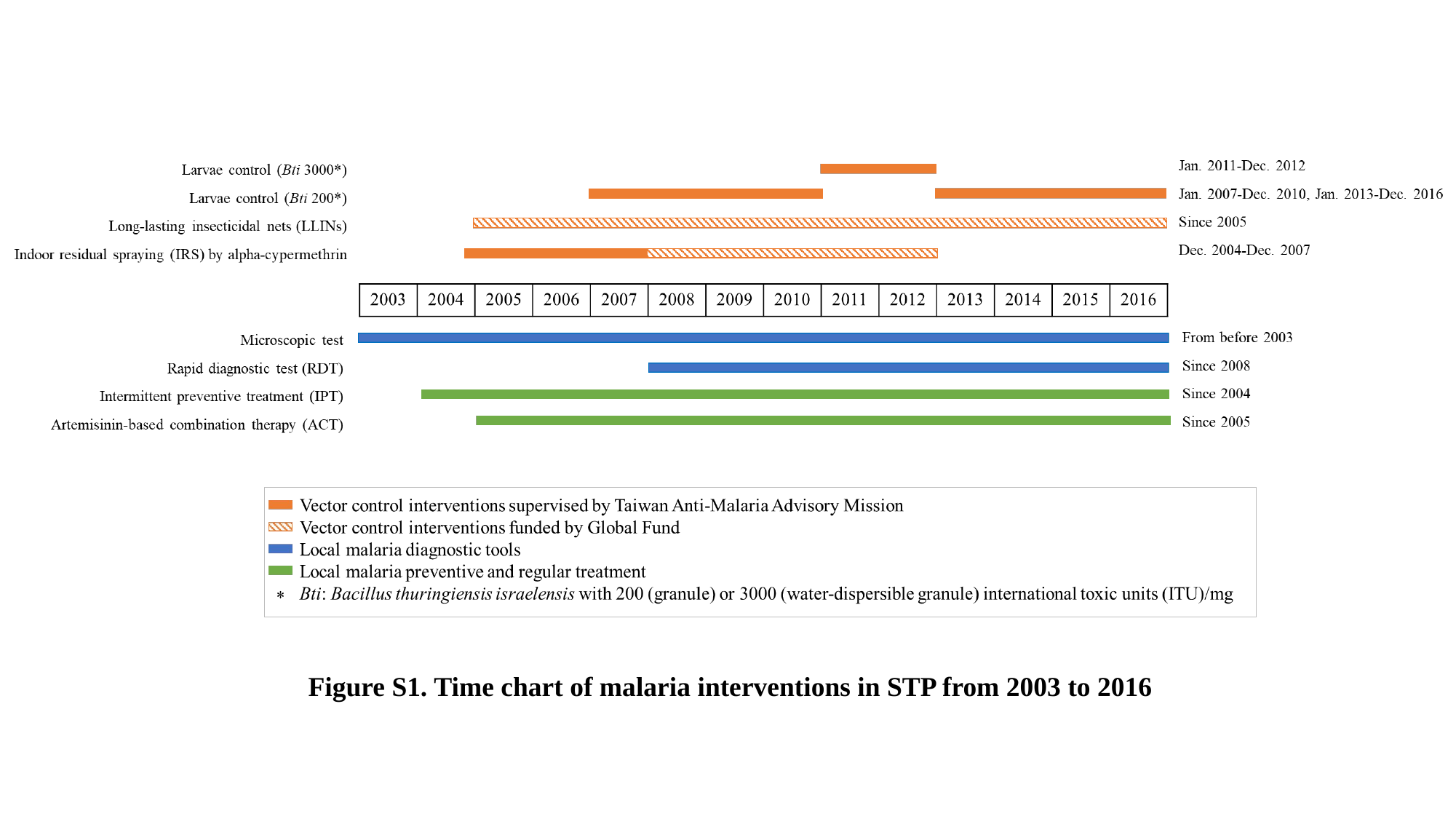

Figure S1. Time chart of malaria interventions in STP from 2003 to 2016

## Slide 2
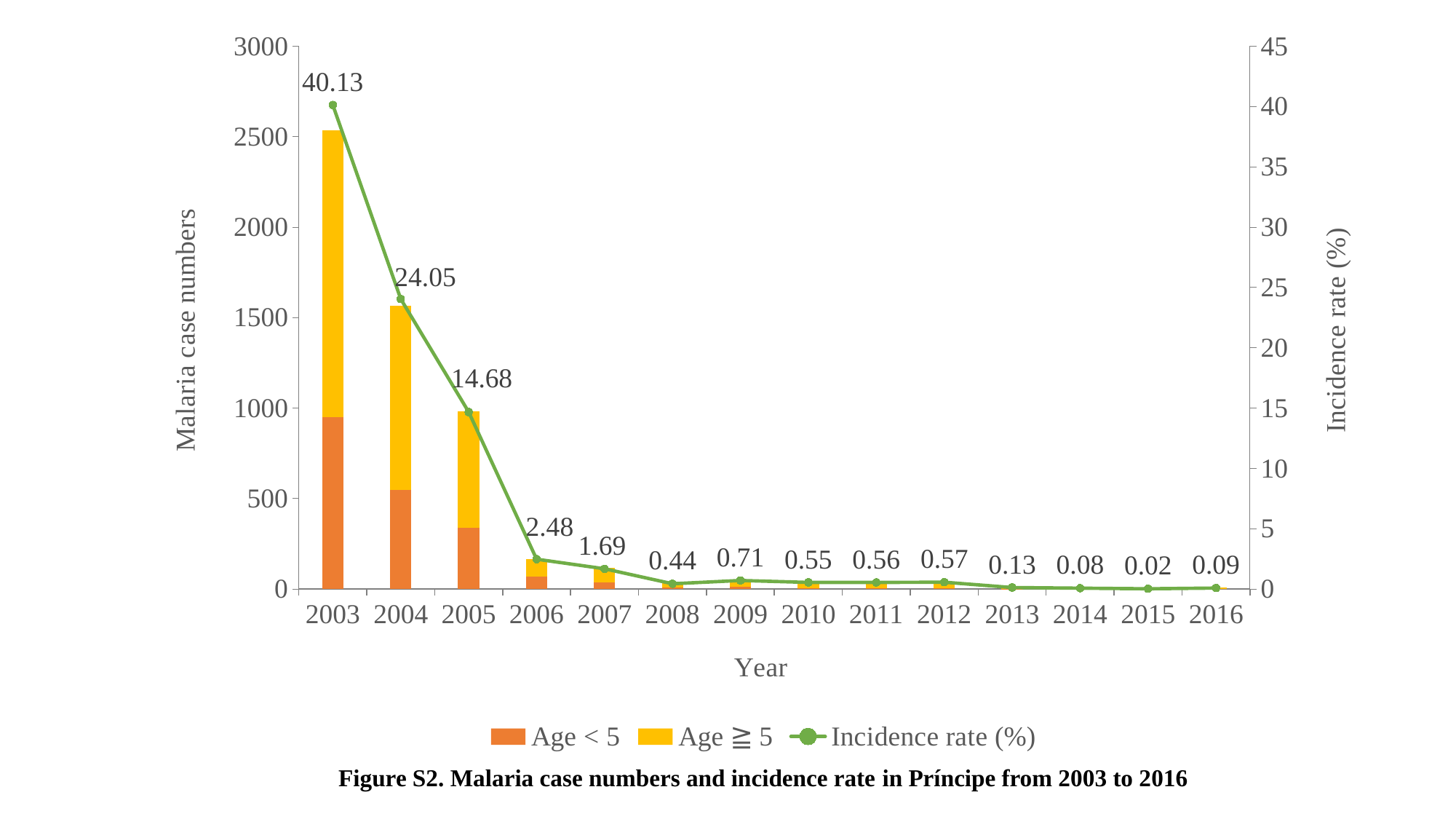

### Chart
| Category | Age < 5 | Age ≧ 5 | |
|---|---|---|---|
| 2003 | 952.0 | 1585.0 | 40.12970578930718 |
| 2004 | 546.0 | 1019.0 | 24.051021976333182 |
| 2005 | 337.0 | 646.0 | 14.678214125727939 |
| 2006 | 70.0 | 97.0 | 2.4788481519964374 |
| 2007 | 35.0 | 81.0 | 1.68506682161534 |
| 2008 | 8.0 | 23.0 | 0.442351598173516 |
| 2009 | 11.0 | 40.0 | 0.7139857202855943 |
| 2010 | 5.0 | 35.0 | 0.5532503457814661 |
| 2011 | 6.0 | 33.0 | 0.5557138785978911 |
| 2012 | 4.0 | 38.0 | 0.5734571272528673 |
| 2013 | 2.0 | 8.0 | 0.13008976193573565 |
| 2014 | 0.0 | 6.0 | 0.0775895512737618 |
| 2015 | 0.0 | 2.0 | 0.024996875390576177 |
| 2016 | 0.0 | 7.0 | 0.08695652173913043 |Figure S2. Malaria case numbers and incidence rate in Príncipe from 2003 to 2016

## Slide 3
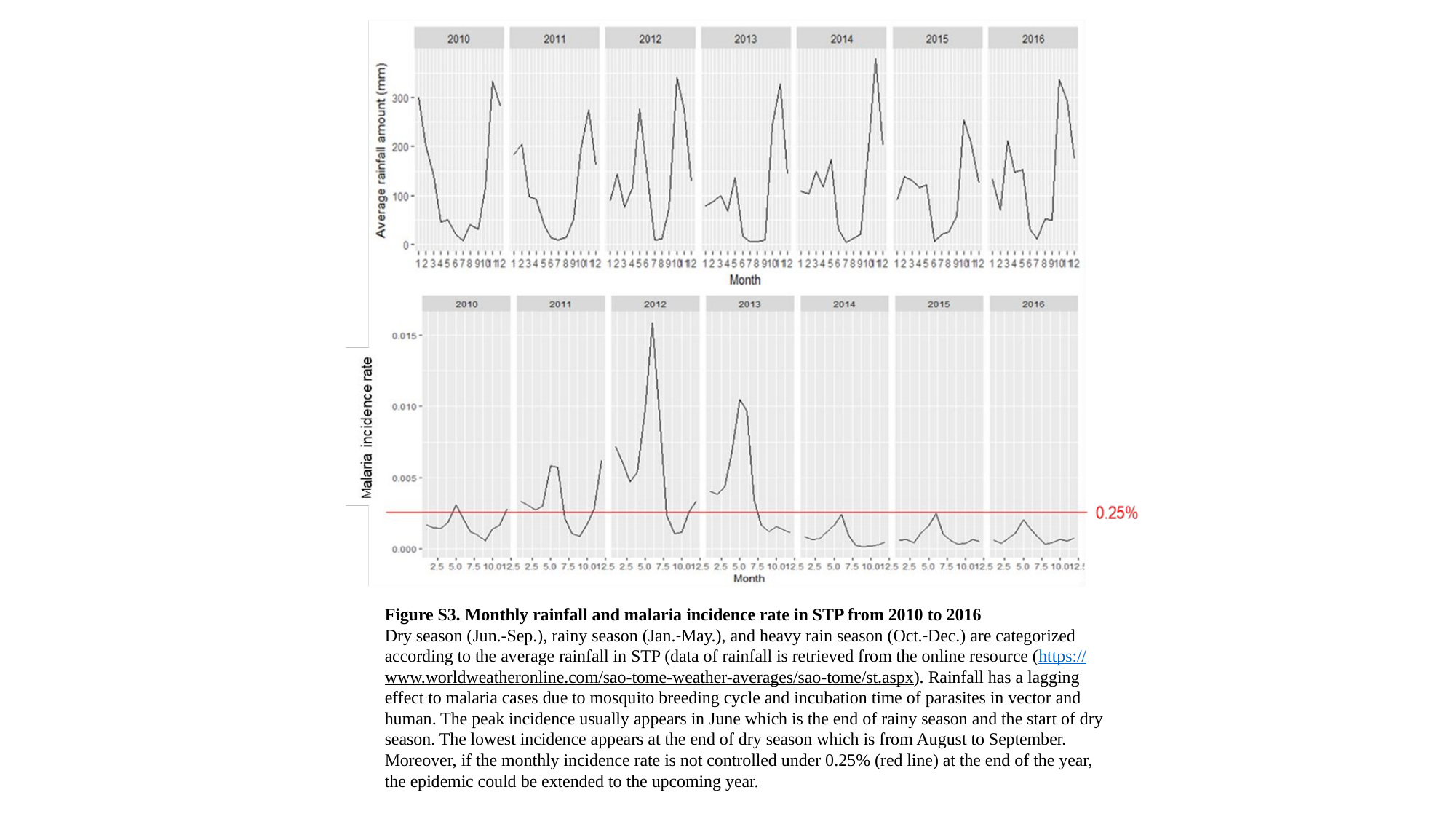

Figure S3. Monthly rainfall and malaria incidence rate in STP from 2010 to 2016
Dry season (Jun.-Sep.), rainy season (Jan.-May.), and heavy rain season (Oct.-Dec.) are categorized according to the average rainfall in STP (data of rainfall is retrieved from the online resource (https://www.worldweatheronline.com/sao-tome-weather-averages/sao-tome/st.aspx). Rainfall has a lagging effect to malaria cases due to mosquito breeding cycle and incubation time of parasites in vector and human. The peak incidence usually appears in June which is the end of rainy season and the start of dry season. The lowest incidence appears at the end of dry season which is from August to September. Moreover, if the monthly incidence rate is not controlled under 0.25% (red line) at the end of the year, the epidemic could be extended to the upcoming year.
