# Supplementary material for: Effects of indoor residual spraying and outdoor larval control on Anopheles coluzzii from São Tomé and Príncipe, two islands with pre-eliminated malaria
Source: Malar J. 2019 Dec 5;18:405. doi: 10.1186/s12936-019-3037-y (PMC6896513; doi:10.1186/s12936-019-3037-y)
Supplement: Supplementary file 2 — Additional file 2: Table S1. COI haplotypes of An. coluzzii in STP. Table S2. Primers for COI PCR. Table S3. Comparisons of mosquito density in different seasons. Table S4. AMOVA analysis of genetic variations in An. coluzzii populations by COI. Table S5. Frequency of kdr L1014F mutation in 1,923 An. coluzzii from 7 districts during 2010 to 2016. Table S6. Nationwide population census in 2012 in STP. [file 12936_2019_3037_MOESM2_ESM.docx]

**Table S1. *COI* haplotypes of *An. coluzzii* in STP**

Two major haplotypes (1, 5) are in bold type. Numbers in parentheses indicate the sequence count if more than 1. Mosquito samples are lacked in MZ in 2010 and 2011, CU in 2010, and PR in 2010, 2011 and 2013. Genbank accession numbers of haplotype 1-39 are MH025842-025880, and haplotype 40-48 are MK330882-330890.

**Table S2. Primers for *COI* PCR**

| Primer | Sequence 5’→3’ | Amplified position in *COI* (without primers) |
| --- | --- | --- |
| LCO | GGTCAACAAATCATAAAGATATTGG | 6-663 |
| HCO | TAAACTTCAGGGTGACCAAAAAATCA |  |
| UEA3 | TATGCATTCCCACGAATAAATAA | 255-925 |
| ACORI | TGTTCCGTGTAATGTAGCTAATC |  |
| ACOFI | GAGCTCATCATATATTTACAGTTG | 845-1506 |
| UEA10 | TCCAATGCACTAATCTGCCATATTA |  |

**Table S3. Comparisons of mosquito density in different seasons**

| Method  Season | Indoor HLCs | | Outdoor HLCs | | Indoor MLTs | | Outdoor MLTs | | Total | |
| --- | --- | --- | --- | --- | --- | --- | --- | --- | --- | --- |
|  | Difference | P _adj._ | Difference | P _adj._ | Difference | P _adj._ | Difference | P _adj._ | Difference | P _adj._ |
| Dry－Heavy rain | -0.10 | **0.0073** | -0.61 | 0.16 | -0.55 | **0.0008** | -1.40 | 0.061 | -0.64 | **0.0007** |
| Dry－Rainy | -0.05 | 0.149 | -0.97 | **0.003** | -0.43 | **0.0054** | -1.36 | **0.037** | -0.67 | **0.00005** |
| Heavy rain－Rainy | 0.04 | 0.322 | -0.36 | 0.50 | 0.13 | 0.65 | 0.04 | 0.998 | -0.03 | 0.98 |

Pairwise comparisons are performed by ANOVA Tukey’s test using R ver 3.5.1. P _adj._ is the p-value adjusted for multiple comparisons. Significant p-values (below 0.05) are highlighted in bold. Dry season is from June to September. Heavy rain season is from October to December. Rainy season is from January to May. HLCs = human landing catches; MLTs = mosquito light traps

**Table S4. AMOVA analysis of genetic variations in *An. coluzzii* populations by *COI***


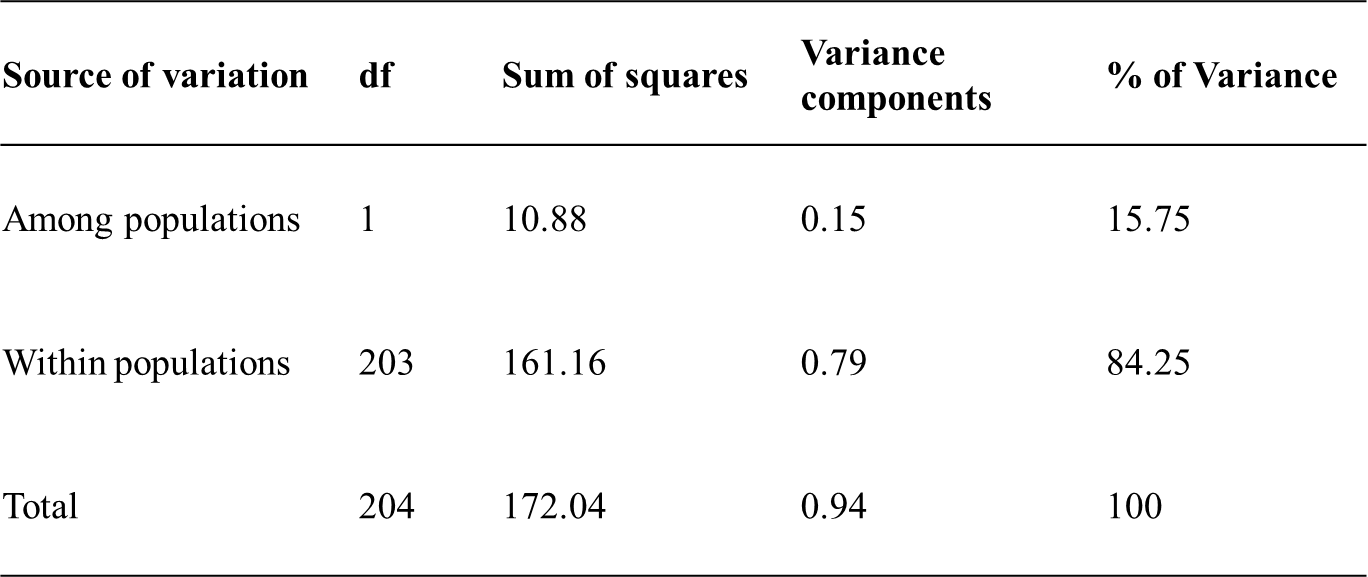


Two populations are *An. coluzzii* from Príncipe and São Tomé, respectively. Mean F_ST_ is 0.16 (p < 0.001).
The major genetic variation in *COI* is from within populations. The AMOVA analysis is performed by Arlequin ver 3.5.

**Table S5. Frequency of *kdr* L1014F mutation in 1,923 *An. coluzzii* from 7 districts during 2010 to 2016**

No. = number of samples. SS = *kdr* 1014 susceptible homozygotes. RR = *kdr* 1014 resistant homozygotes. RS = *kdr* 1014 heterozygotes. The allele frequency and 95% confidence interval (CI) are estimated by Genepop ver 4.2. Mosquito samples are lacked in MZ in 2010-2011, CU in 2010, and PR in 2010, 2011 and 2013.

**Table S6. Nationwide population census in 2012 in STP**

| **District** | **Area (km^2^)** | **Population (%)** | **Population density (population/km^2^)** |
| --- | --- | --- | --- |
| AG | 16.5 | 69,454 (38.86) | 4209.3 |
| MZ | 122 | 44,752 (25.04) | 366.8 |
| LO | 105 | 19,365 (10.83) | 184.4 |
| CT | 119 | 17,161 (9.60) | 144.2 |
| LE | 229.5 | 14,652 (8.20) | 63.8 |
| CU | 267 | 6,031 (3.37) | 22.6 |
| PR | 142 | 7,324 (4.10) | 51.6 |
| **Total** | **1,001** | **178,739** | 178.6 |

Reference: Instituto Nacional De Estatística da Republica Democrática de São Tomé e Príncipe: Publicação dos Resultados sobre Localidades

(<https://www.ine.st/index.php/publicacao/documentos/category/76-dados-localidade-projecoes>)
